# Effects of Astragalus Polysaccharide on Immune Responses of Porcine PBMC Stimulated with PRRSV or CSFV

**DOI:** 10.1371/journal.pone.0029320

**Published:** 2012-01-09

**Authors:** Zeng-Yu Zhuge, Yao-Hong Zhu, Pan-Qi Liu, Xiao-Dong Yan, Yuan Yue, Xiao-Gang Weng, Rong Zhang, Jiu-Feng Wang

**Affiliations:** College of Veterinary Medicine, China Agricultural University, Beijing, China; Queen Elizabeth Hospital, Hong Kong

## Abstract

**Background:**

Astragalus polysaccharide (APS) has been used as an immunomodulator that can enhance immune responses, whereas the immunomodulatory effects of APS on porcine peripheral blood mononuclear cells (PBMCs) exposed to porcine reproductive and respiratory syndrome virus (PRRSV) and classical swine fever virus (CSFV) have not been investigated.

**Methodology/Principal Findings:**

Porcine PBMCs were cultured in complete RPMI media in the presence of the R98-strain of PRRSV (5×10^4^ TCID_50_/ml) or C-strain of CSFV (10^3^ TCID_50_/ml) with or without APS. The expression of mRNA for CD28, cytotoxic T-lymphocyte antigen 4 (CTLA-4), transforming growth factor-β (TGF-β), interleukin 2 (IL-2) and IL-10 was assayed by TaqMan real-time RT-PCR. The expression of mRNA for CD28 and CTLA-4 increased at 24 h after stimulation of PBMCs with CSFV and the increased production of CTLA-4 was confirmed by western blot analysis, whereas the increases were inhibited by the addition of APS. In addition, APS alone upregulated IL-2 and TGF-β mRNA expression in PBMCs and the addition of APS had the capacity to prevent a further increase in IL-2 mRNA expression in PBMCs during CSFV or PRRSV infection, but had no effect on TGF-β mRNA expression. The production of tumor necrosis factor-alpha (TNF-α) increased at 12 h after stimulation with PRRSV or CSFV, but not with PRRSV plus APS or CSFV plus APS, whereas the addition of APS to PBMCs infected with PRRSV or CSFV promoted IL-10 mRNA expression.

**Conclusions:**

We suggested that APS had immunomodulatory effects on cells exposed to PRRSV or CSFV. It might be that APS via different mechanisms affects the activities of immune cells during either PRRSV or CSFV infection. This possibility warrants further studies to evaluate whether APS would be an effective adjuvant in vaccines against PRRSV or CSFV.

## Introduction

Porcine reproductive and respiratory syndrome virus (PRRSV) and classical swine fever virus (CSFV) are both single-stranded RNA viruses that cause highly contagious diseases and lead to tremendous economic losses worldwide [1], [2]. Invasion of PRRSV begins with the inability of the host's anti-viral defenses to control replication of the virus, which arises from evasion of the early warning components of the immune system and leads to long-lasting viremia [3], [4]. Infection with CSFV causes severe leukopenia, particularly of the lymphocytes. The target cells for CSFV in the peripheral blood appear to be mainly monocytes, lymphocytes and granulocytic cells, but all leukocyte populations can be depleted during CSFV infection [5]. Viral infectious diseases are not treated effectively with drugs but are prevented by vaccination with appropriate vaccines. Combined application of a vaccine with an adjuvant or immunopotentiator could improve the efficacy of a vaccine; however, new strains of virus resistant to chemical adjuvants continue to emerge and potent adjuvant action is often correlated with increased toxicity [6].

*Astragalus membranaceus* (AM) is a traditional Chinese medicinal herb used as a tonic to enhance immune defense functions. The antiviral activity of AM is thought to be mainly due to modulatory effects on the immune system. Evidence indicates that AM extract has mitogenic activity on mammalian splenocytes, and is capable of enhancing lymphocyte blastogenesis and stimulating macrophage activation without cytotoxic effects [7], [8]. Astragalus polysaccharide (APS), extracted from AM, has an extensive effect on alleviating immune stress [9], activating the immune system by clearing the immune complex [10], enhancing the transformation of T lymphocytes, and activating B lymphocytes and dendritic cells (DC) [11]–[13]. Several hundred cellular genes have been shown to be altered by AM extract (e.g., *Astragalus*) treatment. Some of these responses are associated with the induction of a cytokine gene profile directed toward a generalized or preparative immune/inflammatory response such as promoting the production of interleukin 2 (IL-2) and interferon-gamma (IFN-γ), and thus improving immune defense functions and resisting the invasion of the external pathogens [14]–[17].

Peripheral blood mononuclear cells (PBMCs) are a heterogenous population of blood cells that include monocyte and lymphocyte immune cells consisting of T-cells, B cells and NK cells. These blood cells represent a critical component in the immune system for fighting infection and adapting to intruders. Since the development of *ex vivo* production of immune cells, PBMCs have emerged as a critical resource for immune responses to PRRSV [18], and CSFV infection is demonstrated to strongly affect the function of PBMCs [19].

CD28 may be considered one of the most important co-stimulatory receptors necessary for T-cell activation [20]. CD28 is constitutively expressed on both naïve and activated T-cells and lowers the T-cell receptor (TCR) activation threshold by binding cognate ligands B7-1/CD80 or B7-2/CD86 on the surface of professional antigen presenting cells (APCs) [21]. Studies examining primary infections with other RNA viruses such as vesicular stomatitis virus and influenza type A virus indicated that CD28 was required for primary expansion of antiviral CD8^+^ T-cells [22], [23].

Cytotoxic T-lymphocyte antigen 4 (CTLA-4) is a structural homolog of the co-stimulatory molecule CD28 and is a negative regulator required for T-cell homeostasis and tolerance [24]. CTLA-4 is about 30% homologous with CD28 and binds to the same ligands as CD28, albeit with a much higher affinity, and CD28 was shown to be important in enhancement of viability and cytokine production by T-cells [25]. This suggests that CTLA-4 preferentially interacts with homologous ligands, and therefore aids in the termination of immune responses activated by CD28 [26]. CD28 and CTLA-4 transduce activation signals that lead to the expression of anti-apoptotic proteins and enhance the synthesis of several cytokines including IL-2 [27].

The tumor necrosis factor (TNF) superfamily is a second group of co-stimulatory molecules. Previous studies have suggested that TNF family members can replace CD28 co-stimulation in primary antiviral CD8^+^ T-cell responses [28], [29]. Cytokines, such as the proinflammatory cytokines TNF-α, IL-2 and IFN-γ and the anti-inflammatory cytokines transforming growth factor-β (TGF-β) and IL-10 are detected during the course of inflammation.

Therefore, the purpose of the present study was to investigate CD28, CTLA-4 and some cytokines involved in immune responses of porcine PBMCs exposed to PRRSV and CSFV with or without APS. These data may help evaluate whether APS could be served as an adjuvant in vaccines against PRRSV or CSFV.

## Results

### PBMC proliferation

**Figure 1:**
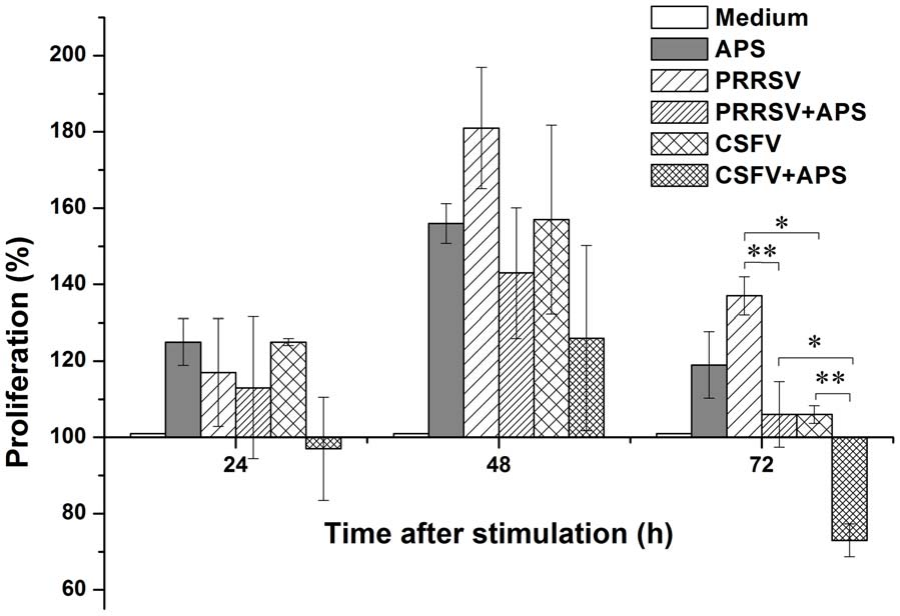
The cell proliferation determined by MTT assay. Porcine PBMCs were cultured with medium alone, APS, PRRSV, PRRSV plus APS, CSFV, or CSFV plus APS. Cell proliferation was assessed after 24, 48 and 72 h of cultivation. The results are presented as a percentage relative to the OD570 values of nonstimulated PBMC (100%) at the same day. Data are presented as means ± SEM of three independent experiments. Within the same time: *, *P*<0.05; **, *P*<0.01.

There were no significant differences in PBMC proliferation among the different stimulation conditions at either 24 h or 48 h after stimulation. At 72 h after stimulation, PBMC proliferation was higher in the presence of PRRSV or CSFV only compared to PRRSV plus APS (*P* = 0.006) or CSFV plus APS (*P* = 0.005), respectively (Figure 1).

### Expression of CD28 and CTLA-4

**Figure 2:**
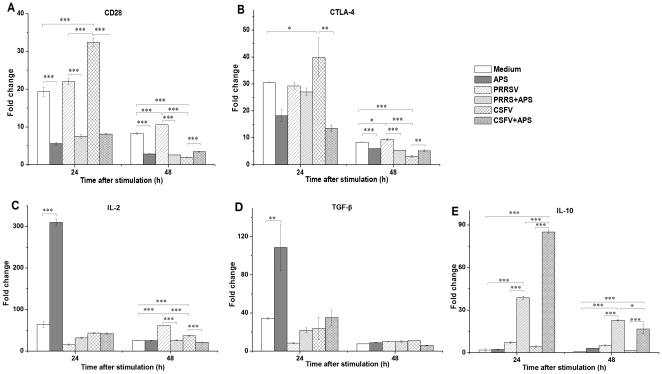
Relative mRNA expression of CD28 (A), CTLA-4 (B), IL-2 (C), TGF-β (D) and IL-10 (E) in porcine PBMCs. Relative mRNA expression in porcine PBMCs cultured with medium alone, APS, PRRSV, PRRSV plus APS, CSFV, or CSFV plus APS. Gene expression was analysed by Taqman real-time RT-PCR. Data are presented as means ± SEM of three independent experiments. Within the same time: *, *P*<0.05; **, *P*<0.01; ***, *P*<0.001.

Compared to medium alone, CD28 mRNA expression increased significantly at 24 h after stimulation with CSFV (*P*<0.001) but not with CSFV plus APS (Figure 2A); in contrast, CD28 mRNA expression decreased (*P*<0.001) at both 24 h and 48 h after stimulation with only APS. Notably, CD28 mRNA expression was higher (*P*<0.001) in the presence of PRRSV alone than in the presence of PRRSV plus APS at 24 h after stimulation. At 48 h after stimulation, the expression of CD28 increased (*P*<0.001) in the presence of PRRSV, but decreased (*P*<0.001) in the presence of CSFV compared to medium alone.

**Figure 3:**
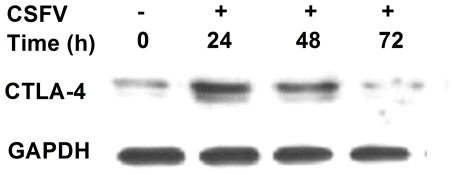
Western blot of CTLA-4 expression in porcine PBMC stimulated with CSFV. Proteins from cell lysate were separated by SDS-PAGE gel and transferred to a nitrocellulose membrane. The membrane was probed with human CTLA-4 affinity purified polyclonal antibody and rabbit anti-goat IgG HRP affinity purified antibody. The GAPDH in each sample was amplified as an internal control as shown in the lower panel. Data are representative of three independent experiments.

Similarly, CTLA-4 mRNA expression increased significantly at 24 h after stimulation with CSFV (*P*<0.045) but not with CSFV plus APS compared to medium alone (Figure 2B). At 48 h after stimulation, the expression of CTLA-4 mRNA was higher (*P*<0.001) in the presence of PRRSV alone than in the presence of PRRSV plus APS, but lower (*P* = 0.003) in the presence of CSFV alone than in the presence of CSFV plus APS. Furthermore, western blot analysis indicated that the production of CTLA-4 increased at 24 h after stimulation with CSFV compared to medium alone (Figure 3).

### Expression of IL-2, TGF-β and IL-10

An increase in IL-2 mRNA expression (*P*<0.001) was detected in the presence of APS only compared to medium alone (Figure 2C). IL-2 mRNA expression increased significantly (*P*<0.001) at 48 h after stimulation with PRRSV or CSFV, but not with PRRSV plus APS or CSFV plus APS compared to medium alone. The expression of TGF-β mRNA increased (*P* = 0.004) at 24 h after stimulation with APS, but neither with PRRSV nor CSFV with or without APS compared to medium alone (Figure 2D). At 48 h after stimulation, there were no significant differences in TGF-β mRNA expression among the different stimulation conditions.

IL-10 mRNA expression increased significantly at 24 h (*P*<0.001) and 48 h (*P*<0.001) after stimulation with PRRSV plus APS or CSFV plus APS, but not with PRRSV or CSFV only compared to medium alone (Figure 2E).

### Production of TNF-α and IFN-γ

**Figure 4:**
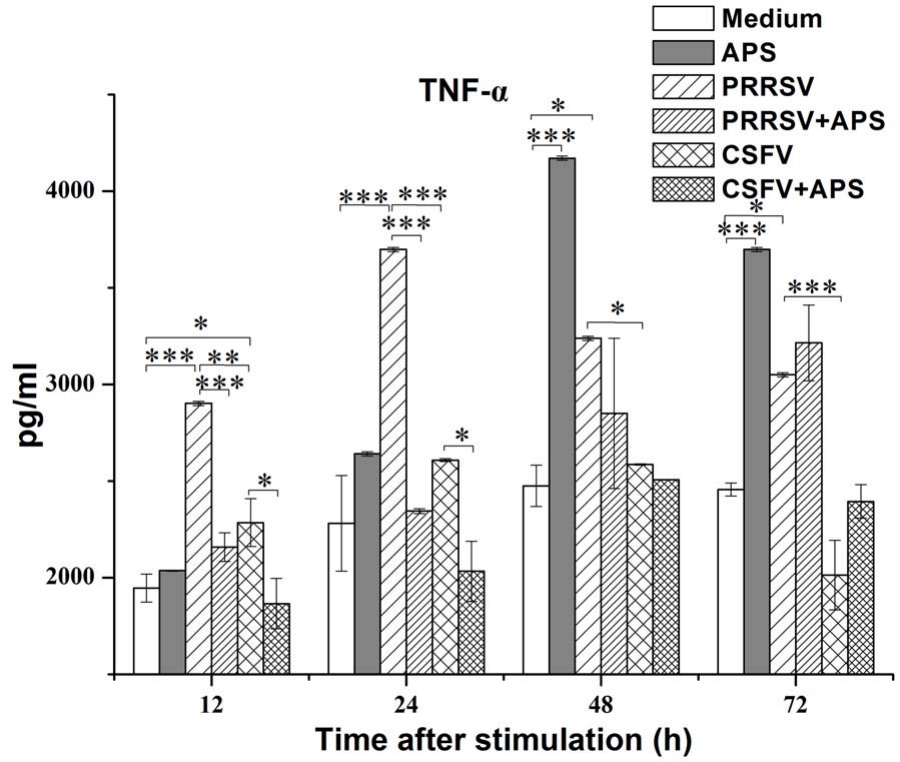
Enzyme-linked immunosorbent assay of TNF-α in cell culture supernatants. Porcine PBMCs were cultured with medium alone, APS, PRRSV, PRRSV plus APS, CSFV, or CSFV plus APS. Data are presented as means ± SEM of three independent experiments. Within the same time: *, *P*<0.05; **, *P*<0.01; ***, *P*<0.001.

The production of TNF-α increased significantly at 12 h after stimulation with PRRSV (*P*<0.001) or CSFV (*P* = 0.030), but not with PRRSV plus APS or CSFV plus APS compared to medium alone (Figure 4). At 24 h after stimulation, the production of TNF-α was even higher in the presence of PRRSV (*P*<0.001) or CSFV (*P* = 0.013) than in the presence of PRRSV plus APS or CSFV plus APS, respectively. Notably, the addition of APS did not significantly increase the production of TNF-α in comparison to medium alone throughout the study.

IFN-γ production in culture supernatants was not detected by ELISA assay.

## Discussion

Effective T-cell responses are crucial for the clearance of viral infections. In some instances, however, the immune response is unable to control viral replication, thereby allowing the virus to persist. The activation and expansion of naïve T-cells requires co-stimulatory signals provided by CD28. CD28 co-stimulation can affect the optimal development of secondary responses, proliferation of memory CD4^+^ and CD8^+^ T-cells and clearance of viral infections [30], [31]. CTLA-4 is a T-cell co-stimulator, which is structurally related to CD28 and exhibits inhibitory activity toward T-cell activation [32], [33]. It has been reported that APS has a dramatic effect on immunologic enhancement and antiviral action such as promoting the expression of CD86, one of the ligands for CD28 and CTLA-4 [34]. We found that the expression of CD28 and CTLA-4 was increased by exposure of cells to CSFV and the increased production of CTLA-4 was confirmed by western blot analysis, whereas the increases were inhibited by the addition of APS. In addition, the addition of APS also decreased CD28 and CTLA-4 mRNA expression in porcine PBMCs infected with PRRSV. Our findings indicated that APS downregulated the expression of CD28 and CTLA-4 in porcine PBMCs infected with CSFV or PRRSV.

IL-2 is a potent T-cell growth factor that induces lymphokine-activated killer activity, mediates activation-induced cell death and is an essential factor for the development of regulatory T-cells (Tregs) [35]. Data presented here unequivocally showed that APS alone increased IL-2 mRNA expression in PBMCs and the addition of APS had the capacity to prevent a further increase in IL-2 mRNA expression in PBMCs during CSFV or PRRSV infection. A previous study indicated that IL-2 did not have the capacity to prevent apoptosis of T-cells from pigs infected with CSFV when tested *in vitro*; in contrast, it sometimes enhanced cell death [5]. Therefore, a possible concept for lymphocyte depletion during CSFV infection might be an aberrant triggering of lymphocytes by an imbalance of virus-induced immune factors or by a viral superantigen.

Tregs can suppress immune responses through the production of immunosuppressive cytokines such as TGF-β and IL-10 [36]. Anti-inflammatory cytokines such as TGF-β and IL-10 specifically inhibit the release of TNF and other proinflammatory mediators [37]. We found that APS had no effect on TGF-β mRNA expression, but increased IL-10 mRNA expression in PBMCs infected with PRRSV or CSFV. It may be a multistep process in which increased IL-10 initially induces the suppressive environment that may prevent the induction of IL-2 and TNF-α. Interleukin-10 is identified as one of the postulate mechanisms for immunomodulation, both systemically and locally, during an early stage of PRRSV infection [38], [39]. Recent data suggest that the prevalence of IL-10 responses to PRRSV was higher in vaccinated animals than in naïve pigs [40]. On the other hand, the increased IL-10 production during persistent viral infection induces T-cell inactivation and results in the prevention of viral clearance [41], [42].

The synthesis and release of proinflammatory cytokines often represent changes in immune responses during the course of CSF, and TNF-α may be one of the most important proinflammatory mediators in the pathogenesis of the disease. An infection of monocytes by CSFV could induce TNF-α secretion [43]. We showed that primed porcine PBMCs could produce TNF-α in response to PRRSV or CSFV. Notably, TNF-α production was higher in PBMCs infected by PRRSV than by CSFV. It has been shown that TNF-α exerts a strong inhibitory effect on the transcription of the CD28 gene [44]. This may be an explanation for the increased CD28 mRNA expression in PBMCs exposed to CSFV, but not in PBMCs exposed to PRRSV.

IFN-γ has been thought to play a crucial role against infectious viruses. There is a positive correlation between IFN-γ responses induced by vaccination and resistance to the abortifacient effects of PRRSV and the addition of IFN-γ inhibited PRRSV replication in macrophages [45]. Previous studies have shown that PRRSV infection failed to elicit any significant inflammatory cytokine expression as an initial response [46], [47]. The production of IFN-γ in supernatant was not detected in our study. Although the significance of a relatively low IFN-γ response during PRRSV infection relating to protective immunity is unknown, this may allow for the establishment of persistent infection of PRRSV in pigs.

In conclusion, the present data indicated that APS alone upregulated IL-2 and TGF-β mRNA expression in PBMCs. The addition of APS to PBMCs infected with PRRSV or CSFV had no effect on TGF-β mRNA expression, downregulated the expression of mRNA for CD28, CTLA-4 and IL-2, but promoted IL-10 mRNA expression. Thus, we suggested that APS had immunomodulatory effects on cells exposed to PRRSV or CSFV. It might be that APS via different mechanisms affects the activities of immune cells during either PRRSV or CSFV infection. This possibility warrants further studies to evaluate whether APS would be an effective adjuvant in vaccines against PRRSV or CSFV.

## Materials and Methods

### Virus and Astragalus polysaccharides

The R98-strain of PRRSV and the rabbit propagated C-strain of CSFV were kindly provided by Dr. Baoshou Yang (Ruipu Company, Hebei, China). Freeze-dried APS was kindly provided by Prof. Fenghua Liu (Beijing University of Agriculture, Beijing, China).

### Animals and blood sampling

Six twelve-week-old crossbred pigs, free of PRRSV- and CSFV-specific antibodies and antigens, were obtained from a commercial farm. The use and care of all animals in this study was approved by the China Agricultural University Animal Ethics Committee under the protocol (CAU-AEC-2010-038). Blood was collected from the anterior vena cava and anti-coagulated with 0.1 volume of 2×acid–citrate–dextrose (0.15 M sodium citrate, 0.076 M citric acid monohydrate, 0.287 M dextrose) solution.

### PBMC isolation

PBMCs were prepared by Ficoll gradient centrifugation according to the manufacturer's instructions. Briefly, 10 ml of Ficoll–Hypaque (Sigma, St. Louis, MO) was stratified under 20 ml of peripheral blood and centrifugation was performed at 400 ×*g* for 20 min at room temperature. Recovered PBMCs were washed three times with D-PBS (2.67 mM KCl, 1.47 mM KH_2_PO_4_, 137.93 mM NaCl, 8.06 mM Na_2_HPO_4_·7H_2_O). The number of living cells was counted by trypan blue (Sigma, St. Louis, MO) staining at a concentration of 0.4 mg/ml under the microscope. For all of the following experiments, freshly isolated PBMC were used.

### Cell cultures

Cells were cultured in RPMI-1640 culture medium (Invitrogen, Carlsbad, CA) containing 2 mM L-glutamine enriched with 100 U/ml penicillin/streptomycin (Sigma, St. Louis, MO) and 10% heat-inactivated fetal calf serum and were counted to determine the PBMC cell number with an equal volume of trypan blue under the microscope. The cell concentration was brought to 2×10^6^ cells/ml culture medium in the presence of PRRSV (5×10^4^ TCID_50_/ml), CSFV (10^3^ TCID_50_/ml) or APS (10 µg/ml). Cells in the virus groups (PRRSV or CSFV) were allocated to the non-APS and APS (10 µg/ml) groups. Cells and supernatants were harvested at 0, 12, 24, 48 and 72 h after stimulation and stored at −80°C until further analysis.

### MTT assay

Cells were cultured at 2×10^6^ cells/well and cell proliferation was measured by MTT (3-(4,5-dimethylthiazol-2-yl)-2,5-diphenyl tetrazolium bromide) (Sigma, St. Louis, MO) colorimetric assay at 24, 48 and 72 h after stimulation. Ten µl of MTT filtered stock solution (5 mg/ml) was added to 200 µl of cells and incubated for 4 h at 37°C. After incubation, 100 µl 0.1 M HCl in absolute isopropanol was added to each tube. The optical density (OD) at a wavelength of 570 nm was measured using an enzyme-linked immunosorbent assay plate reader (Bio-Rad, Hercules, CA). Results are presented as % over nonstimulated PBMC (100%) at the same day.

### Total RNA extraction

Total RNA was extracted from PBMC using Trizol reagent (Invitrogen, Carlsbad, CA). The final RNA was eluted in an appropriate amount of RNase-free water (Qiagen, Valencia, CA). For each sample, the integrity of RNA extracted was analyzed by agarose gel electrophoresis by staining with ethidium bromide and visualization under UV light. The amount of RNA extracted was determined and its purity (OD260/OD280 absorption ratio >1.9) was verified using a NanoDrop® ND-2000C Spectrophotometer (NanoDrop Technologies Inc., Wilmington, DE).

### Quantitative real-time RT-PCR

Quantitative real-time RT-PCR was performed using a 7500 Real-Time PCR System (Applied Biosystems, Foster City, CA). For each sample, 600 ng of total RNA were added directly to a TaqMan one-step RT-PCR reaction containing rTth DNA polymerase enzyme (Applied Biosystems, Foster City, CA). The thermal cycling profile of this assay consisted of a 25 min reverse transcription step that was performed at 42°C for 5 min, then 60°C for 20 min, 2 min of Taq polymerase activation at 95°C, followed by 45 cycles of PCR at 95°C of denaturing for 15 s and 60°C of annealing/extension for 1 min. A non-template control of nuclease-free water was included in each run. All reactions were conducted in triplicate.

IL-10, TGF-β and hypoxanthine phosphoribosyl-transferase (HPRT) were assayed using published sequences of primers and probes [48], which are listed in Table 1. Probes were dual-labeled with 6-carboxyfluorescein (FAM) as the 5′-reporter and 3′ TAMRA quencher (Applied Biosystems, Foster City, CA). The commercially available Taqman Gene Expression Assays (Applied Biosystems, Foster City, CA): *IL2* (Ss03392429_m1), *CD28* (Ss03373720_s1), *CTLA-4* (Ss03394185_m1) were used in the present study. Each system was re-evaluated and optimized for the current approach, and all reagents were titrated for optimal performance using total RNA isolated from porcine PBMCs that were induced to produce the various cytokines and receptors according to established protocols. Five-fold serial dilutions (triplicate) of total RNA extracted from PBMCs were amplified by real-time RT-PCR using gene-specific primers to calculate the amplification efficiency of each system and a non-template control of nuclease-free water was included in each run.

**Table 1:** Sequences of oligonucleotide primers and probes used for TaqMan real-time RT-PCR, the length of the PCR products and accession numbers.

Gene specificity	Oligonucleotide sequences (5′-3′) of primers and probes		Product length (bp)	GenBank No. in NCBI site
IL-10^a^	F	CGGCGCTGTCATCAATTTCTG	89	L20001
	R	CCCCTCTCTTGGAGCTTGCTA		
	Probe	FAM-AGGCACTCTTCACCTCCTCCACGGC-TAMRA		
TGF-β1^a^	F	TACGCCAAGGAGGTCACCC	156	NM-214015
	R	CAGCTCTGCCCGAGAGAGC		
	Probe	FAM-CTAATGGTGGAAAGCGGCAACCAAATCTA-TAMRA		
HPRT^a^	F	GTGATAGATCCATTCCTATGACTGTAGA	104	U69731
	R	TGAGAGATCATCTCCACCAATTACTT		
	Probe	FAM-ATCGCCCGTTGACTGGTCATTACAGTAGCT-TAMRA		
CD28^b^			82	AY435219.1
CTLA-4^b^			82	NM-214149.1
IL-2^b^			152	NM-213861.1

IL-10, interleukin 10; TGF-β1, transforming growth factor-beta1; HPRT, hypoxanthine phosphoribosyl-transferase; CTLA4, cytotoxic T-lymphocyte antigen 4; F, forward primer; R, reverse primer. 5′and 3′modifications for the probes were as followed: FAM, 6-carboxyfluorescein; TAMRA, tetramethylrhodamine;

a [48].

b Taqman Gene Expression Assays (Applied Biosystems, Foster City, CA).

The cycle threshold (Ct) value was determined for each sample. To evaluate the relative quantification of mRNA expression, the Ct-values of the target genes were normalized to the Ct-values of the housekeeping gene HPRT and the results were presented as fold change using the 2^−ΔΔCt^ method.

### Protein quantification by ELISA

Concentrations of TNF-α and IFN-γ in cell culture supernatants were determined by means of commercially available ELISA kits (R&D Systems, Minneapolis, MN). The dynamic range of the TNF-α assay was 23.4 to 1500 pg/ml. The minimum detectable dose was 3.7 pg/ml. The intra-assay coefficient of variation was <4.2% and the inter-assay coefficient of variation was <6.5%. The dynamic range of the IFN-γ assay was 2.7 to 11.2 pg/ml. The mean minimum detectable dose was 6.1 pg/ml. The intra-assay coefficient of variation was <2.9% and the inter-assay coefficient of variation was <9.4%.

### Western blot

PBMCs were harvested and lysed in lysis buffer containing 150 mM NaCl, 10 mM Tris-HCl (pH 7.9), 0.5% Triton X-100, 0.6% NP-40, supplemented with protease inhibitors (1 µg/ml leupeptin, 1 µg/ml pepstatin A, and 2 µg/ml aprotinin). Lysates were frozen at −80°C, thawed, and centrifuged to remove the insoluble pellet. The protein concentration in the supernatant was determined by a protein assay (Bio-Rad Laboratories, Hercules, CA). Sodium dodecylsulfate-polyacrylamide gel electrophoresis (SDS-PAGE) sample buffer (10 mM Tris-HCl, pH 6.8, 2% SDS, 10% glycerol, 0.2 M DTT) was added to the lysates. Lysates were heated to 100°C for 5 min, and 80 µg of protein was loaded into each well of a 10% SDS-PAGE gel. Resolved proteins were electrophoretically transferred to nitrocellulose and blocked with 5% non-fat milk, and the primary antibody human CTLA-4 affinity purified polyclonal antibody (dilution 1∶500). After overnight incubation at 4°C the blots were washed, exposed to secondary antibody rabbit anti-goat IgG HRP (horseradish peroxidase) (R&D Systems, Minneapolis, MN) (dilution 1∶1000) for 1 h, and finally detected by ECL. The GAPDH (glyceraldehyde-3-phosphate dehydrogenase) in each sample was amplified as an internal control as shown in the lower panel.

### Statistical analysis

All statistical evaluations were conducted by use of the SPSS statistical software, Version 16.0 (SPSS Inc.,Chicago, IL). The data were compared using the parametric test one-way analysis of variance (ANOVA) and differences were tested by non-parametric Dunnett's test. *P*-values <0.05 were considered statistically significant. The data were presented as means ± SEM.
